# Upregulation of Circulating PD-L1/PD-1 Is Associated with Poor Post-Cryoablation Prognosis in Patients with HBV-Related Hepatocellular Carcinoma

**DOI:** 10.1371/journal.pone.0023621

**Published:** 2011-09-01

**Authors:** Zhen Zeng, Feng Shi, Lin Zhou, Min-Na Zhang, Yan Chen, Xiu-Juan Chang, Yin-Ying Lu, Wen-Lin Bai, Jian-Hui Qu, Chun-Ping Wang, Hong Wang, Min Lou, Fu-Sheng Wang, Ji-Yun Lv, Yong-Ping Yang

**Affiliations:** 1 Center of Therapeutic Research for Hepatocellular Carcinoma, Beijing 302 Hospital, Beijing, China; 2 Research Center for Biological Therapy, Beijing 302 Hospital, Beijing, China; 3 Institute of Microbiology, Chinese Academy of Sciences, Beijing, China; National University of Singapore, Singapore

## Abstract

**Background:**

The programmed cell death-1 receptor/programmed cell death-1 ligand (PD-1/PD-L1) pathway plays a crucial role in tumor evasion from host immunity. This study was designed to evaluate the association between circulating PD-L1/PD-1 and prognosis after cryoablation in patients with HBV-related hepatocellular carcinoma (HCC).

**Methodology/Principal Findings:**

In the present study, 141 HBV-related HCC patients were enrolled and of those 109 patients received cryoablation. Circulating PD-L1/PD-1 expression was tested by flow cytometry, and 23 patients were simultaneously evaluated for intratumoral PD-L1 expression by immunohistochemical staining. Circulating PD-1/PD-L1 expression was associated with severity of diseases in patients with HCC, and the circulating PD-L1 expression was closely correlated with intratumoral PD-L1 expression. Of the clinical parameters, PD-1/PD-L1 expression was associated with tumor size, blood vessel invasion and BCLC staging. Moreover, PD-1/PD-L1 expression dropped after cryoablation while being elevated at the time of tumor recurrence. Patients with higher expression of circulating PD-L1, as well as circulating PD-1, had a significantly shorter overall survival and tumor-free survival than those with lower expression. Multivariate analysis confirmed that circulating PD-L1 could serve as an independent predictor of overall survival and tumor-recurrence survival in HCC patients after cryoablation.

**Conclusions/Significance:**

Upregulation of circulating PD-L1/PD-1 is associated with poor post-cryoablation prognosis in patients with HBV-related hepatocellular carcinoma.

## Introduction

Hepatocellular carcinoma (HCC) is a complex condition with multiple variables affecting the disease course and response to treatment, including liver function and performance status of the patient and tumor stage [1], [2]. Patients with hepatitis B or hepatitis C virus infection are also at a higher risk of developing HCC, and over 85% of patients with HCC present with HBV infection in China [3]. Surgical treatment options for patients with HCC include resection and liver transplantation[4], [5]. Local ablation, such as cryoablation like surgery, is also considered as a potentially curative therapy [6]. This technique has the advantages of being minimally invasive, exerting fewer effects on liver function, and shows better reproducibility and improved immunity following treatment as compared with traditional surgical approaches. Our previous study [7] indicate that cryoablation not only directly destroys the malignant tissues, but also exerts effects on the tissue adjacent to the carcinoma. Yantorno et al. [8] and Shulman et al. [9] have postulated that cryoablation interferes with the biological activity of tumor cells while preserving the structure of tumor antigenic proteins, which may enhance the specific anti-tumor immune response. Sabel et al. [10], [11] used cryoablation in BALB/c mice with MT-901 mammary adenocarcinoma tumors and reported that cryoablation led to the induction of both a tumor-specific T-cell response in the tumor-draining lymph node and increased systemic NK cell activity. These observations were correlated with tumor rejection upon re-challenge in mice that had undergone cryoablation. Osada et al. [12] performed cryoablation in 13 HCC patients with unresectable tumors. Following treatment, not only was the local tumor found to be necrotic, but the adjacent tumor tissue was also necrotic and shrunken, which was regarded as ectopic tumor suppression. This response may be associated with the release of tumor antigens, resulting in host production of anti-tumor antibodies [13].

Programmed cell death-1 receptor (PD-1), a novel co-inhibitory receptor mainly expressed on activated T and B cells [14], belongs to the CD28 family, with 28% identity to the extracellular region of CTLA-4 [15], [16]. Programmed cell death-1 ligand (PD-L1, also known as B7-H1), the ligand of PD-1, can be induced in monocytes, dendritic cells, and parenchymal cells under the stimulation with proinflammatory cytokines, such as type-I and type-II interferons [17]. There is growing evidence to show that PD-L1 can deliver an inhibitory signal to PD-1 expressing T cells, leading to suppression of the immune response by inducing apoptosis, anergy and functional exhaustion of T cells, which subsequently contributes to the compromised tumor immunity [18]. Until now, the relationship between intratumoral PD-L1 and tumor aggressiveness, and clinicopathological features as well as overall survival has been well described in several human malignancies, such as ovarian, esophageal and pancreatic cancer [19]-[21]. A recent report demonstrated that HCC patients with higher expression of intratumoral PD-L1 had a significantly poorer prognosis than that of HCC patients with lower expression in the overall survival time after resection. [22]. Our previous report [23] showed PD-1 and PD-L1 upregulation promotes CD8^+^T-cell apoptosis and post-operative recurrence in HCC patients. However, the detection of intratumoral PD-L1 requires an invasive operation for liver biopsy and would be a complex test for clinical applications, and there have been few reports on changes in circulating PD-L1/PD-1 levels in patients with HCC before and after cryoablation. Therefore, the present study was designed to investigate the association between cryoablation and circulating PD-L1/PD-1 variation in patients with HCC and to explore the role of circulating PD-L1/PD-1 in the prognosis of HCC.

## Materials and Methods

### Patients

In total, 141 patients with HBV-related HCC were enrolled in this study from January 2006 to January 2009. Based on the Barcelona Clinic Liver Cancer (BCLC) staging classification [24], 46 patients were classified as stage A, 63 as stage B,and 32 as stage C (Table 1). Except for the patients at stage C, the 109 remaining patients received curative treatment with complete cryoablation lesions initially by dynamic spiral computed tomography or contrast-enhanced dynamic magnetic resonance imaging and were followed-up. All 109 patients had received no prior treatment such as chemotherapy or radiation therapy. Individuals with concurrence of autoimmune disease, HIV and syphilis were excluded. The decision whether to include these patients into this study was made by a multidisciplinary team of doctors coming from different departments. The study protocol was approved by the Beijing 302 Hospital Research Ethics Committee, and written informed consent was obtained from the patients who met the inclusive criteria prior to blood and tumor sampling. The primary follow-up endpoint of the study was tumor-free survival time (TFS). The secondary endpoint was overall survival time(OS). TFS was calculated from the date of commencement of cryoablation to the date of recurrence HCC. OS was calculated from the date of commencement of cryoablation to the date of death or last follow-up.

**Table 1:** Basic clinical features of 141 patients with hepatitis B-related HCC.

Clinical factor	Stage A	Stage B	Stage C
Cases	46	63	32
Age (years)	53.1(35–68)	52.9(28–70)	52.8(36–71)
Male/Female	33/13	45/18	31/1
Tumor diameter (cm)	2.1(0.5–3)	4.5(3–6)	6.8(5–9)
AFP level≥20<20	24/22	40/23	22/10
HBV DNA (-/<10^5^≥10^5^)	20/12/14	17/22/24	10/16/6
Child-Pugh (A/B)	33/13	42/21	20/12

HCC, hepatocellular carcinoma; AFP, α-fetoprotein; HBV, hepatitis B virus.

### Argon–helium cryoablation and sample collection

Argon–helium cryoablation was followed as our previously described [25]. Briefly, argon-helium gas based CRYOcare system (EndoCare,Irvine, CA, USA) and cryoprobes were used to freeze the tumor with a dual freeze-thaw cycle under ultrasound-guiding. The size and number of probes depended on the location and size of the lesions to be ablated. A single 15-minute freeze was performed, followed by a passive thaw. The iceball dimensions were monitored via intraoperative ultrasonography. Cryoprobe temperatures were reduced in 1 minute to −135°C±2°C. We carried out ablation of all tumors with a curative intent, with a margin of 1 cm beyond the edge of the tumor if feasible, in a single cryoablation. The procedure was performed with the patient under conscious sedation. Liver function tests, α-fetoprotein (AFP) detection, and abdominal computed tomography (CT) or magnetic resonance imaging (MRI) were performed regularly. Anti-coagulated blood (heparin) was obtained 1 day before treatment and 1, 4weeks after treatment. A singleaction Biopsydevice (Promex Technologies, Franklin, IN, USA) was used during the operation with ultrasound guidance, and biopsy of the tumor tissue was performed in 23 patients.

### Flow cytometric analysis

Antibodies conjugated with different fluoresceins, including fluorescein isothiocyanate (FITC), phycorerythrin(PE), peridin chlorophyll protein (PerCP) and allophycocyanin (APC) were used for flow cytometric analysis. PE-conjugated anti-PD-1, PD-L1 and APC-conjugated anti-CD14 were purchased from eBiosciences (San Diego, CA,USA); PerCP-conjugated anti-CD8 and APC-conjugated anti-CD3 were purchased from BD Biosciences (San Jose, CA,USA). In detail, PE-conjugated anti-PD-L1 and APC-conjugated anti-CD14 were applied for the PD-L1 detection on monocytes; PE-conjugated anti-PD-1, PerCP-conjugated anti-CD8 and APC-conjugated anti-CD3 were used for the PD-1 detection on CD8+T cells. To determine of positive expressions of PD-1 and PD-L1, PE-conjugated IgG_1_ (eBiosciences) were adopted in this assay. For each test, 50 µl fresh heparinized peripheral blood was incubated with indicated antibodies (20 µl) for 20 min and then lysed with FACS^TM^ lysing solution (BD Biosciencs, San Jose, CA, USA), washed with PBS, fixed and analyzed by the fluorescence activated cell sorting (FACS) analysis (FACS Calibur, BD Biosciences, San Jose, CA, USA). Data were analyzed by Flowjo software (TriStar, San Carlos, CA, USA).

### Immunohistochemical staining for PD-L1 evaluation in liver tissues

Based on the availability within all 141 HCC patients, there were twenty-three patients enrolled in this study by simultaneously collecting peripheral blood and liver tumor biopsy, which included 9 stage A, 8 stage B and 6 stage C patients in detail. Paraffin-embedded, formalin-fixed liver tissue from these enrolled HCC patients was cut into 5-µm sections and placed on polylysine-coated slides. Antigen retrieval was achieved via pressure cooking for 10 minutes in citrate buffer. Mouse monoclonal anti-human PD-L1 antibody (Biolegend, San Diego, CA, USA) was used. 3-amino-9-ethyl-carbazole (red color) was used as a substrate followed by counterstaining with hematoxylin. For the evaluation of PD-L1 staining, three independent pathologists examined the slides without access to clinical information. The intensity of the PD-L1 staining was scored as one of four degrees according to the area of positive staining, including score 0, less than 25%; score 1, 25%–50%; score 2, 50%–75%; and score 3, higher than 75%. The results for score 0 and 1 were considered as low PD-L1 expression, whereas score 2 and 3 were considered as high PD-L1 expression, accordingly.

### Statistical analysis

Data were summarized and presented as means ± SD and analyzed using SPSS13.0 software (SPSS Inc, Chaicago, IL,USA). The Mann–Whitney nonparametric U-test was applied for comparisons between 2 groups and the Kruskal–Wallis nonparametric H-test was performed for comparisons among multiple groups. The χ2 test was used for comparison of the individual variables. The Wilcoxon matched-pairs test was used to compare the data from the same individuals. Receiver operating characteristic (ROC) curve analysis was used to determine the OS predictive value of the parameters, and the differences in the area under the curve (AUC) were detected by using SPSS13.0.Tumor recurrence and survival rates were analyzed by the Kaplan–Meier method. The log-rank test was applied to compare findings among groups. The Cox regression model was used to perform univariate and multivariate analyses. A value of P<0.05 was considered to be statistically significant.

## Results

### Upregulated circulating PD-L1/PD-1 expression is related to HCC progression

**Figure 1:**
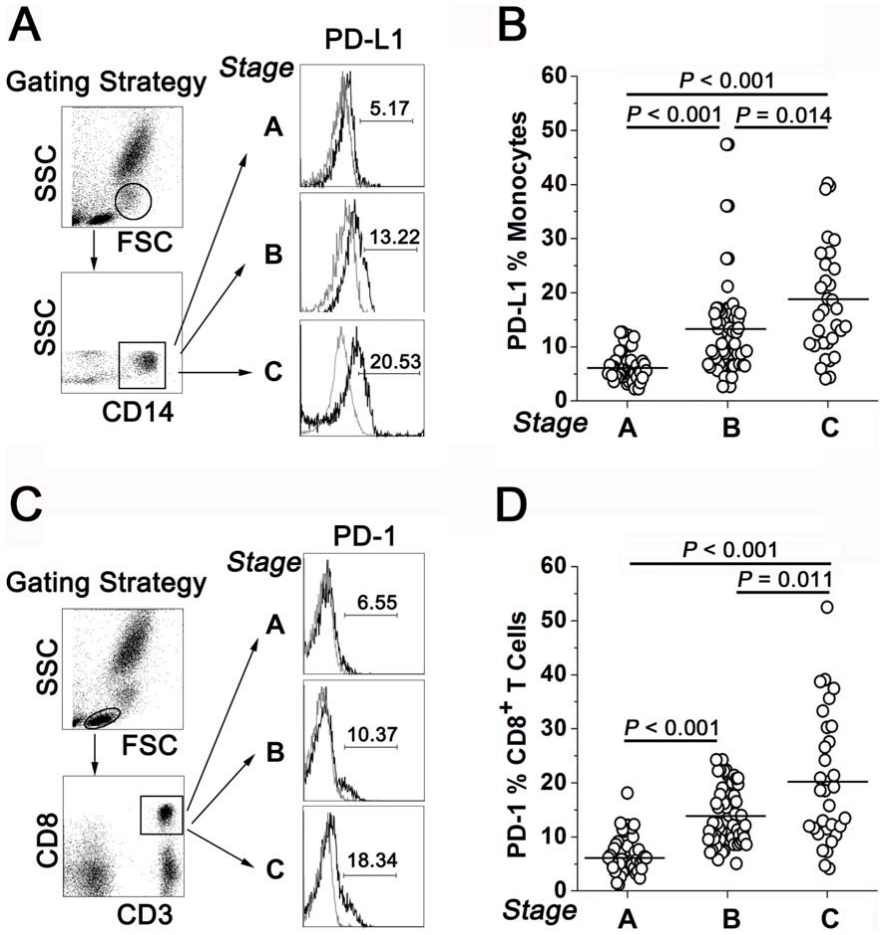
Gating strategies and expression profiles of circulating PD-L1 and PD-1 in 141 patients with HBV-related HCC. The gating strategies and representative results of circulating PD-L1 (A) and PD-1 (C) expression are shown. FSC, forward scatter; SSC, side scatter. In the histogram, the black line indicates PD-L1 or PD-1, whereas the grey line represents its corresponding isotype control. Numbers in the histograms show the frequencies of positive cells. The statistical results for circulating PD-L1 (B) and PD-1 (D) were organized according to the HCC stages (stage A, n = 46; stage B, n = 63; stage C, n = 32), respectively. The black bar indicates the mean value.

We firstly investigated the circulating PD-L1/PD-1 expression by using flow cytometric analysis in a cohort of 141 HCC patients. The gating strategies for both types of expression are shown (Fig. 1A and 1C). Our results revealed that the circulating PD-L1 expression in monocytes increased from stage A to stage B (Fig. 1B; vs. stage A, *P*<0.001) and reached the highest level at stage C (Fig. 1B; vs. stage A, *P*<0.001; vs. stage B, *P* = 0.014). Moreover, the circulating PD-1 exhibited a similar expression profile to PD-L1 (Fig. 1D; stage B vs. stage A, *P*<0.001; stage C vs. stage A, *P*<0.001; stage C vs. stage B, *P* = 0.011). Therefore, circulating PD-1/PD-L1 expression was associated with the disease severity in patients with HCC.

**Figure 2:**
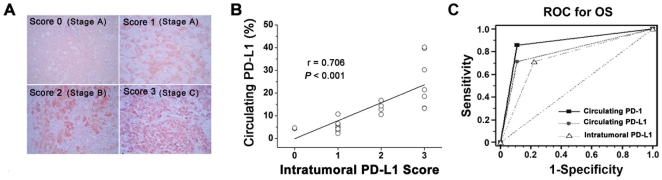
Circulating PD-L1 expression was closely correlated with their corresponding intratumoral PD-L1 expression. (A)Representative immunohistochemical results of PD-L1 for different degrees are shown(red for PD-L1, ×400). (B) Statistical analysis demonstrated that circulating PD-L1 was significantly correlated with intratumoral PD-L1(r = 0.706, P<0.001). (C) The predictive ability of circulating PD-L1/PD-1 compared with that of intratumoral PD-L1 by receiver operating characteristic (ROC) curves for overall survival (OS).

As a previous report demonstrated that intratumoral PD-L1 was significantly associated with overall survival in HCC patients [22], we compared the expression levels between intratumoral and circulating PD-L1. According to the staining area and intensity, we scored the intratumoral PD-L1 expression as one of four levels of degree, from score 0 to score 3 (Fig. 2A). Within a total of 23 HCC patients, we found circulating PD-L1 expression was closely correlated with their corresponding intratumoral PD-L1 expression (Fig. 2B; r = 0.706, P<0.001). We also noticed a tendency for the intratumoral PD-L1 expression that the stage A patients are preferentially showing a score 0-1, compared with score 3 for stage C patients (Fig. 2A). Furthermore, ROC curves were used to compare that intratumoral PD-L1 and circulating PD-1/PD-L1, and to determine which one was a better parameter to predict the OS prognosis of the HBV-associated liver cancer patients. The results showed that although the area of intratumoral PD-L1(0.80±0.10, 95%CI:0.58–O.94) was higher than that of circulating PD-L1(0.75±0.11, 95%:0.52–0.90,P = 0.55) and lower than circulating PD-1 (0.87±0.08, 95%;0.67–0.97,P = 0.56), there were no statistic difference(Fig. 2C).

### Correlations between circulating PD-1/PD-L1 expression and clinical parameters in patients with HCC

Patients with HCC (n = 141) were divided into 2 groups according to the median of circulating PD-1/PD-L1 expression: those with low PD-1/PD-L1 expression (<10.88%/9.18) and those with high PD-1/PD-L1 expression (≥10.88%/9.18). Upon analysis, tumor size, blood vessel invasion and BCLC staging were associated with PD-1/PD-L1 expression. However, no significant relationships were found between PD-1/PD-L1 expression and gender, age, number of tumors, HBV DNA load, AFP level or Child-Pugh class (Table 2).

**Table 2:** Correlation between circulating PD-L1/PD-1 expression and clinical features of 141 HCC patients.

	PD-L1			PD-1		
Characteristics	Low	High	P	Low	High	P
Age (years)						
≥54	38	35	0.346	35	38	0.945
<54	30	38		33	35	
Gender						
Male	50	59	0.302	50	59	0.179
Female	18	14		19	13	
AFP (ng/ml)						
≥20	40	46	0.610	38	48	0.105
<20	28	27		32	23	
Tumor size (cm)						
≥3	32	63	≤0.001	31	64	≤0.001
<3	36	10		39	7	
Tumor number						
Single	43	39	0.238	47	35	0.051
Multiple	25	34		24	35	
Child-Pugh						
A	48	47	0.432	48	47	0.764
B	20	26		22	24	
Vascular invasion						
Yes	6	26	0.001	7	25	0.001
No	60	49		63	46	
HBV DNA (U/ml)						
negative	21	26	0.925	27	20	0.314
<10^5^	24	26		21	29	
≥10^5^	20	24		22	22	
BCLC staging			≤0.001			≤0.001
Stage A	36	10		38	8	
Stage B	26	37		25	38	
Stage C	6	26		7	25	

X^2^ tests for all analysis.

### Dynamic circulating PD-1/PD-L1 variation after cryoablation

**Figure 3:**
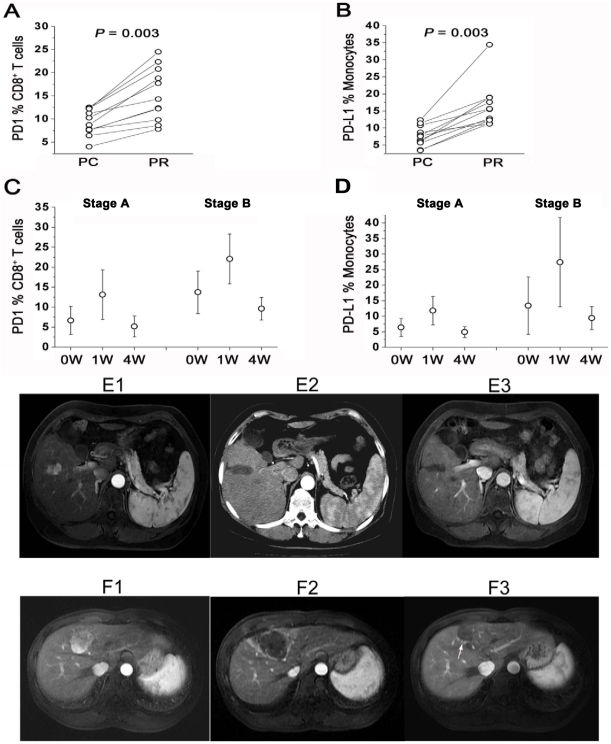
Dynamic circulating PD-1/PD-L1 variation after cryoablation in HCC patients. (A,B) HCC patients with postoperative recurrent tumors (n = 11, PR, post-recurrence)showed that circulating PD-1/PD-L1 expression individually increased 4 weeks after cryoablation (PC, post-cryoablation). (C,D)Circulating PD-1/PD-L1 expression in 109 HCC patients increased significantly 1 week after cryoablation when compared with that before treatment, and dropped 4 weeks later. (E)A HCC patient(stageA, E1) receiving cryoablation showed tumor completely necrosis one week later(E2) and the necrotic tumor tissue shrank 33 months later with no tumor recurrence(E3). (F) A HCC patient(stageB, F1) receiving cryoablation showed tumor completely necrosis one week later(F2) and tumor recurred at the margin of necrotic tumor tissue 18 months later(F3, arrow indicates the recurred tumor).

The results of the analyses showed that circulating PD-1/PD-L1 expression in 109 HCC patients increased significantly 1 week after cryoablation when compared with that prior to treatment, and liver function impairment was observed at 1 week after treatment. The measure of liver function reached normal within 4 weeks after treatment (Table 3), accompanied by decreased PD-1/PD-L1 expression (Fig. 3C,3D). In addition, studies of patients with postoperative recurrent tumors (n = 11) revealed that the prevalence of circulating PD-1/PD-L1 individually increased compared with the periods without tumors(4weeks after cryoablation) (Fig. 3A, 3B, E, F).

**Table 3:** Hepatic function of 109 HCC patients before and after cryoablation.

Weeks	ALB (g/L)	ALT (U/L)	AST (U/L)	TBIL (mol/L)	PA (%)
Baseline	38.75±4.31	42.33±22.14	48.25±16.17	20.38±12.17	87.08±12.31
1	35.52 ±3.35*	96.71±25.32*	102.32±20.38*	26.78±13.12 *	80.58±13.1*
4	37.54±6.32	38.52±15.41	44.12±18.23	18.11±8.92	86.2±11.32

* : *P*<0.05 *versus* baseline.ALB, albumin; ALT, alanine aminotransferase; AST aspartate aminotransferase; TBIL, total bilirubin; PA, prothrombin activity.

### Increased prevalence of circulating PD-1/PD-L1 predicts poor prognosis in HCC patients after cryoablation

**Figure 4:**
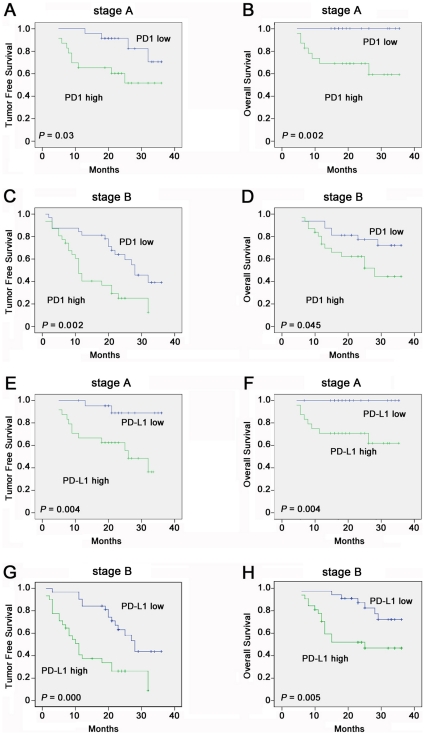
Increased expression of circulating PD-L1/PD-1 predicts poor prognosis in HCC patients after cryoablation. 109 HCC patients were divided into 2 groups by the median value of circulating PD-1/PD-L1 at the time before treatment as low PD-1 (n = 23, 4.05±1.28% for stageA; n = 31, 9.26±1.63% for stage B) and high PD-1 (n = 23, 9.26±3.09% for stage A; n = 32, 18.05±3.78% for stage B), low PD-L1 (n = 23, 4.26+0.93% for stageA; n = 31, 7.17±2.19% for stage B) and high PD-L1 (n = 23, 8.51+2.48% for stage A; n = 32, 19.37±9.41% for stage B). (A,B)HCC patients in stage A with lower expression of circulating PD-1 have longer TFS and OS than those with higher expression(P = 0.03 and P = 0.002,respectively). (C,D) HCC Patients in stage B with lower expression of circulating PD-1 have longer TFS and OS than those with higher expression(P = 0.002 and P = 0.045,respectively). (E,F)HCC patients in stage A with lower expression of circulating PD-L1 have longer TFS and OS than those with higher expression(P = 0.004 and P = 0.004,respectively). (G,H)HCC patients in stage B with lower expression of circulating PD-L1 have longer TFS and OS than those with higher expression(P = 0.000 and P = 0.005,respectively).

For the 109 patients followed up for a median of 23 months(6∼36months) after the cryoablation, the 1-, 2- and 3-year survival rate was 86%, 75% and 67%, respectively. Furthermore, the 1-, 2-and 3-year recurrence-free survival rate was 70%, 58% and 40%, respectively.To address whether increased circulating PD-1/PD-L1 was associated with prognosis in HCC patients after cryoablation, 46 HCC patients at stages A and 63 HCC patients at stage B were divided into 2 groups by the median value of circulating PD-1/PD-L1 at the time before treatment as low PD-1 (n = 23, 4.05±1.28% for stageA; n = 31, 9.26±1.63% for stage B) and high PD-1 (n = 23, 9.26±3.09% for stage A; n = 32, 18.05±3.78% for stage B), low PD-L1 (n = 23, 4.26+0.93% for stageA; n = 31, 7.17±2.19% for stage B) and high PD-L1 (n = 23, 8.51+2.48% for stage A; n = 32, 19.37±9.41% for stage B). The results showed that the high PD-1/PD-L1 group had a significantly higher rate of tumor recurrence and progression after cryoablation in comparison to the low PD-1/PD-L1 group (Fig. 4). The univariate analysis (Table 4) showed that the overall survival was associated with PD-1 and PD-L1. The recurrence-free survival was related to tumor size, PD-1 and PD-L1. Multivariate analysis (Table 5) using the Cox hazard model revealed that the circulating PD-L1 expression was independent poor prognostic factors for TFS and OS, and PD-1 expression was independent poor prognostic factor for TFS.

**Table 4:** Univariate analyses of prognosis factors associated with survival in 109 HCC patients of stage A/B.

Variables	TFS		OS	
	HR(95%CI)	P	HR(95%CI)	P
Age, y(≥54 vs <54)	0.709(0.446∼1.126)	0.145	0.757(0.436∼1.314)	0.322
Gender(male vs female)	0.853(0.504∼1.442)	0.552	0.743(0.388∼1.423)	0.371
AFP, ng/ml(≥20 vs <20)	1.182(0.739∼1.891)	0.485	1.225(0.694∼2.161)	0.484
Tumor size, cm(≥3 vs <3)	2.033(1.232∼3.353)	0.005	1.654(0.915∼2.991)	0.096
Tumor number(≥2 vs 1)	1.363(0.853∼2.175)	0.195	1.576(0.910∼2.730)	0.105
Child-Pugh(A vs B)	0.858(0.522∼1.410)	0.545	0.694(0.373∼1.290)	0.248
HBV DNA, U/ml(negative vs <10^5^ vs ≥10^5^)	0.961(0.729∼1.267)	0.779	0.832(0.592∼1.168)	0.287
PD-1(low vs high)	2.683(1.666∼4.322)	≤0.001	2.710(1.498∼4.901)	0.001
PD-L1(low vs high)	4.290(2.607∼7.059)	≤0.001	3.180(1.755∼5.759)	≤0.001

Note: Cox proportional hazards regression model.

**Table 5:** Multivariate analyses of prognosis factors associated with survival in 109 HCC patients of stage A/B.

Variables	TFS		OS	
	HR(95%CI)	P	HR(95%CI)	P
Tumor size, cm(≥3 vs <3)	0.744(0.390∼1.423)	0.372	0.740(0.366∼1.495)	0.402
PD-1(low vs high)	1.811(1.011∼3.245)	0.046	1.948(0.96∼3.955)	0.065
PD-L1(low vs high)	3.891(2.151∼7.041)	≤0.001	2.650(1.300∼5.401)	0.007

Note: Cox proportional hazards regression model.

## Discussion

The interaction between PD-1 and PD-L1 has been demonstrated to negatively regulate T-cell activation and functions, leading to inhibition of the immune responses in cancer patients [16], [26], [27]. In addition, patients with PD-L1-positive cancer cells were reported to have a significantly poorer prognosis than those with PD-L1-negative cancer cells in pancreatic cancer, urothelial cancer, breast and ovarian cancer patients [16], [18], [21], [28]. Two recent reports demonstrated that PD-L1 expressed in tumor cells or peritumoral activated monocytes contributed to tumor aggressiveness and postoperative recurrence in hepatocellular carcinoma patients [22], [29]. These investigations indeed provided new evidences for disease diagnosis and prognosis evaluation. However, the invasive collection for liver biopsy makes the whole procedure much complex and inconvenient. Therefore, it seemed worth evaluating the clinical significance of circulating PD-L1 in predicting the disease progression.

In the present study, we detected PD-L1/PD-1 expression on circulating peripheral blood mononuclear cells(PBMCs) and found PD-L1/PD-1 expression increased with liver tumor progression. Further study revealed that there was a close correlation between the circulating and intratumoral PD-L1 expression. We analyzed the circulating PD-1/PD-L1 expression and the clinical parameters in patients with HCC. The result demonstrated that tumor size, blood vessel invasion and BCLC staging were associated with PD-1/PD-L1 expression. However, no significant relationships were found between PD-1/PD-L1 expression and gender, age, number of tumors, HBV DNA load, AFP level or stage of liver function. Although several publications demonstrated that PD-1/PD-L1 expression levels correlated with the HBV DNA titers [30], [31], we did not find any correlations in the present study regarding HCC patients with chronic HBV infection. Besides monocytes, PD-L1 is also expressed in dendritic cells and our previous investigations also revealed a good correlation for PD-L1 expression between these two kinds of cells [23]. However, we abandoned the dendritic cell-associated PD-L1 in the present study due to its more complex detection procedures and more expensive cost.

Our observations suggested that elevated PD-1/PD-L1 expression during the first week after cryoablation was related to inflammation caused by the therapy and that postoperative fever and liver function impairment were the main manifestations. A previous study reported that inflammation could promote PD-1/PD-L1 expression [30]. Subsequent reduction of PD-1/PD-L1 expression 4 weeks after therapy was associated with a recovery from stress and the reduction of tumor burden. Additionally, numerous tumor antigens are released from these necrotic cells, resulting in an immune response and reduction in immune tolerance [8], [9]. Interestingly, Campbell et al [32] found that cryopreservation of PBMC led to a marked reduction of PD-1 and PD-L1 expression in CD3+/CD8+ T cells and CD45+/CD14+ monocytes, with no significant effect on CD3+CD4+ T cells. The previous study [22], [23] suggested that intratumoral PD-L1 was closely associated with the recurrence or metastasis of HCC after surgery, and we compared the circulating PD-1/PD-L1 expression before and after tumor recurrence in 11 HCC patients received cryoablation.The result showed that PD-1/PD-L1 expression was elevated after tumor recurrence. In an *in vitro* study, Chen J et al [33] found that hepatoma cells up-regulate expression of programmed cell death-1 in T cells.Based on the dynamic circulating PD-1/PD-L1 variation after cryoablation and Chen's finding [33], we support the mechanism that PD-1/PD-L1 upregulation may be due to cytokine stimulations in the tumor microenvironment [29].

Growing investigations demonstrate that PD-1 extensively up-regulated on tumor Ag-specific T cells in cancer patients and play a crucial role in the mechanism of tumor evasion by inhibiting the proliferation, cytokine-secretion and cytoxicity of tumor Ag-specific T cells [34]-[36]. Especially, Our previous study show that the interaction between PD-1 and intratumoral PD-L1 promote the apoptosis of CD8+ T cells, which probably contribute to the low expression of tumor Ag-specific T cells in patients with hepatocellular carcinoma [23]. There are two main pathways promoting PD-1 expression on CD8+T cells. One is by antigen specific stimulation; the other is by the cytokine pathway, through which PD-1 upregulations are promoted on non-tumor Ag-specific T cells. Here, we propose that the upregulation of PD-1 on non-tumor Ag-specific T cells should constrain the tumor-associated inflammation to a much milder degree, which hereafter favor the tumor differentiation and proliferation. However, further investigations should be carried out to elucidate the detailed mechanism of this issue.

In the present study, we also found patients with recurrent tumors, which would generally be smaller than primary ones, associated with higher PD-1/PD-L1 expression. According to former investigations, we believe that it is the tumor-associated inflammation, rather than tumor volume, which promote the elevated expression of PD-1 and PD-L1. After cryoablation therapy, tumor cells were degenerated, disaggregated and up-taken by antigen presenting cells, e.g. kuffer cells, pDC and mDC, which lead to the secretion of a large mount of proinflammatory cytokines, including IFN-α,IFN-γ and gamma-chain cytokines. In detail, interferons could promote PD-L1 expression on both tumor cells and antigen presenting cells in comparison with that gamma-chain cytokines account for PD-1 elevations on CD8+T cells. Thus, both PD-1 and PD-L1 expression was significantly up-regulate at the first week post-cryoablation. Together with the resolution of tumor burden, the immune system goes in to a quiescent phase (4w after cryoablation), manifesting lower PD-1 and PD-L1 expression. When recurrence occurs, the immune system should be re-activated and promote an inflammation, both of which favor PD-1 and PD-L1 re-elevation, in comparison with the expression at week 4 post cryoablation therapy, at the time when patients are free of tumor burden.

Recurrence after hepatectomy or ablation is one of main biological features of HCC. Tumor diameter,portal vein tumor thrombus, microvessel invasion, and tumor capsule invasion are the main high risk factors for early recurrence [37]. Identifying the high risk associated with early recurrence contributes to the grasp of operation indications and guidance for adjunctive therapy. The cryoablation approach is similar in therapeutic efficacy and recurrence rate to hepatic resection and radiofrequency ablation (RFA) [38], [39]. In the present study, we found that the high PD-1/PD-L1 group had a significantly higher tumor recurrence or progression rate after cryoablation compared with the low PD-1/PD-L1 group. As the patients received cryoablation were at stage A and stage B, so the well-established factors including tumor vascular invasion and TNM stage were excluded from multivariate analyses.Since the sample size of the present study was limited,further studies are needed to draw valid conclusion for the prognostic factors. Moreover, the multivariate analyses showed PD-L1 seemed to be a much stronger predictor than PD-1, regarding that PD-L1 is the independent predictor for both TFS and OS in comparison to PD-1 for TFS only. Mechanically, it is not easy to explain the difference between PD-L1 and PD-1 on this issue. However, we propose that PD-L1 should be much more sensitive to take changes according to the inflammatory intensity, due to its innate role to protect host from overwhelming inflammation through engagement with PD-1. Thus, the PD-L1 expression seems to be more accurate to reflect the inflammatory changes which are associated with tumor progression.

Many mechanisms have been proposed for an attenuated immune response to tumors, including partial antigen masking, failure in antigen processing, suppression of effector cells and inadequate costimulation [40]-[42]. In the current study, we showed that HBV-related HCC patients with increased PD-1/PD-L1 expression were at significantly increased risk of overall mortality. These results were consistent with the opinion that the PD-1/PD-L1 pathway might, at least in part, contribute to the profile of immunosuppression observed in patients with HCC. These findings also confirmed that PD-1/PD-L1 was one of the immunoinhibitory targets for tumor cell.

Taken together, our study suggested that upregulation of circulating PD-1/PD-L1 could serve as novel valid immunological markers in predicting disease progression of HCC patients after cryoablation; and this would be helpful for the improved clinical management and the development of new therapeutic options for patient with HBV-related HCC. We postulate that the combination of cryoablation and immunotherapy such as anti-PD1 antibody [43] may provide a better prognosis for patients with HCC.
